# Cerebral malaria is associated with long-term mental health disorders: a cross sectional survey of a long-term cohort

**DOI:** 10.1186/s12936-016-1233-6

**Published:** 2016-03-31

**Authors:** Richard Idro, Angelina Kakooza-Mwesige, Benjamin Asea, Keron Ssebyala, Paul Bangirana, Robert O. Opoka, Samson K. Lubowa, Margaret Semrud-Clikeman, Chandy C. John, Joyce Nalugya

**Affiliations:** Department of Paediatrics and Child Health, Mulago Hospital, Makerere University College of Health Sciences, P.O Box 7072, Kampala, Uganda; Neuropediatric Research Unit, Astrid Lindgren Children’s Hospital, Karolinska Institutet, Solna, Stockholm Sweden; Department of Pediatrics, University of Minnesota, Minneapolis, DE USA; Departments of Pediatrics, Microbiology and Immunology, Indiana University School of Medicine, Indianapolis, USA

**Keywords:** Behaviour, Mental health, Psychiatric, Disorder, Children, Cerebral malaria

## Abstract

**Background:**

Cerebral malaria (CM) and severe malarial anaemia (SMA) are associated with neuro-developmental impairment in African children, but long-term mental health disorders in these children are not well defined.

**Methods:**

A cohort of children previously exposed to CM (n = 173) or SMA (n = 99) had neurologic assessments performed and screening for behaviour difficulties using the Strengths and Difficulties Questionnaire (SDQ) a median of 21 months after the disease episode. These findings were compared to concurrently recruited community children (CC, n = 108). Participants with SDQ total difficulties score ≥17 had a mental health interview with the child and adolescent version of the Mini-International Neuropsychiatric Interview (MINI-KID) and a sample had brain magnetic resonance imaging (MRI).

**Results:**

Fifty-five children had SDQ score ≥17. On the MINI-KID, these children were classified as having no difficulties (n = 18), behaviour difficulties only (n = 13) or a mental health disorder (n = 24). Behaviour difficulties were seen in similar frequencies in CM (3.5 %), SMA (4.0 %) and CC (2.8 %). In contrast, mental health disorders were most frequent in CM (10.4 %), followed by SMA (4.0 %) and CC (1.8 %). Externalizing disorders (conduct, oppositional defiance and attention deficit hyperactivity) were the most common mental health disorders. The median total coma duration was 72 (IQR 36.0–115.0) h in patients with mental health disorders compared to 48 (IQR 28.5–78.7) h in those without, *p* = 0.039. Independent risk factors for mental health disorder included neurologic deficit at discharge (OR 4.09 (95 % CI 1.60, 10.5) and seizure recurrences during hospitalization, (OR 2.80, 95 % CI 1.13, 6.97). Brain MRI findings consistent with small vessel ischaemic neural injury was seen in over half of these children.

**Conclusions:**

Cerebral malaria may predispose children to mental health disorders, possibly as a consequence of ischaemic neural injury. There is urgent need for programmes of follow-up, diagnosis and interventions for these children.

## Background

Malaria is a leading cause of ill health, neuro-disability and death in tropical countries. In 2013, the World Health Organization estimated that there were 198 million clinical cases resulting in 584,000 deaths [1]. The clinical manifestations range from asymptomatic parasitaemia to severe and fatal disease. The syndrome of severe malaria is defined by the presence of *Plasmodium falciparum* malaria parasites in peripheral blood and clinical or laboratory evidence of severe vital organ dysfunction [2]. Among African children, severe malaria anaemia, malaria with respiratory distress/metabolic acidosis and malaria with impaired consciousness or coma encompass the majority of severe disease [3].

Over the past 15 years, it has become clearer that many survivors of cerebral malaria (severe malaria with coma) sustain brain injury, and 25 % have long-term neurologic and cognitive deficits [4, 5]. More recently, this research group documented a similar but narrower range of cognitive deficits after severe malaria anaemia [6]. There have also been reports of behaviour difficulties and mental health disorders in cerebral malaria exposed children. These included inattentiveness, hyperactivity, impulsive, and aggressive behaviour, which disrupts normal childhood development [7, 8]. However, there is a paucity of cohort-based longitudinal studies of these problems; the burden of these disorders, the pathogenesis, and treatment needs are poorly understood. It is also unknown whether the difficulties follow other forms of severe malaria. The hypothesis was that, in addition to neurologic and cognitive deficits, cerebral malaria and the other forms of severe malaria predispose children to mental health disorders and that children younger than five years on exposure are at most risk.

The study examined a cohort of children with exposure to cerebral malaria or severe malaria anaemia to assess for mental health disorders and performed magnetic resonance imaging (MRI) on a subset of these children to assess associated structural changes in the brain. In addition, the study also assessed whether specific clinical features predicted mental health disorders.

## Methods

### Design

The study was performed at Mulago Hospital, Kampala, Uganda, as a sub-study within a larger study of neuro-cognitive impairment in children with severe malaria from April 2012 to December 2014. In this study, children with cerebral malaria, severe malaria anaemia, or community children were enrolled if they were between 18 months and 12 years of age. Cerebral malaria was defined as: (1) coma (Blantyre Coma Score [(BCS) ≤ 2]; (2) *Plasmodium falciparum* on blood smear; and (3) no other known cause of coma (e.g., meningitis, a prolonged postictal state or hypoglycaemia-associated coma reversed by glucose infusion). Severe malarial anaemia was defined as presence of *P. falciparum* on blood smear in children with a haemoglobin level ≤5 g/dL. Children with cerebral malaria or severe malaria anaemia were managed according to the Ugandan Ministry of Health treatment guidelines at the time of the study. These included intravenous quinine treatment followed by artemisinin combination therapy if the child could take orally. All underwent a medical history and physical examination. Children with cerebral malaria were assessed for malaria retinopathy [9] by indirect ophthalmoscopy. Clinical laboratory testing was performed as previously described [6]. Patients with a haemoglobin <5 g/dL received a blood transfusion.

Community children were recruited from the nuclear family, extended family, or household compound area of children with cerebral malaria or severe malaria anaemia. Eligible controls were ages 18 months to 12 years and currently healthy. Exclusion criteria for all children included: (1) known chronic illness requiring medical care; (2) known developmental delay; or (3) prior history of coma, head trauma, hospitalization for malnutrition, or cerebral palsy. Additional exclusion criteria for children with severe malaria anaemia included: (1) impaired consciousness on physical exam; (2) other clinical evidence of central nervous system disease; or (3) more than one seizure prior to hospitalization. Additional exclusion criteria for community children included: (1) illness requiring medical care within the previous 4 weeks; or (2) major medical or neurological abnormalities on screening physical exam.

### Recruitment

Children in the main study were asked to participate in the present study of behaviour difficulties and mental health disorders. An earlier preliminary study, documented that behaviour difficulties after cerebral malaria developed within 6 months of exposure [7]. Thus, only children from the main study who had completed at least 6 months follow up, were considered for this study. All eligible children with cerebral were invited, along with a random sample of 115 children with severe malaria anaemia and 115 community children. A sample size of 175 children with cerebral malaria, 88 with severe malaria anaemia and 88 community children was estimated to have >80 % power to demonstrate a two-fold increase in the risk of behaviour or mental health disorders in children with cerebral malaria as compared to severe malaria anaemia or community children. Ethical approval was granted by the Institutional Review Boards for human studies at Makerere University School of Medicine and the University of Minnesota and written consent was obtained from parents or guardians of study participants.

### Screening for behaviour difficulties and mental health disorders

Each child underwent screening for behaviour difficulties using the Strengths and Difficulties Questionnaire (SDQ) [10]. The SDQ is a 25 item behaviour screening tool that comprises five subscales (emotional, conduct, hyperactivity or peer problems, and a pro-social scale) each with five items. Scores from these items are used to generate the total difficulties score (range 0 to 40), which is the sum of scores from all the subscales except the pro-social scale. A total difficulties score of ≥17 is interpreted as abnormal behaviour. Similar cut off levels are used to define abnormalities in each subscale. The SDQ was previously validated in neighbouring Democratic Republic of Congo which has similar socio-cultural settings with Uganda [11]. The parent’s version of the questionnaire was used. The English language version of the questionnaire was translated into Luganda, the most widely used language in central Uganda, and parents could choose to use either version.

### Diagnosis of behaviour difficulty or mental health disorder

Children with SDQ total difficulties score ≥17 were invited for a detailed mental health interview using the Mini-International Neuro-Psychiatric Interview for Children and Adolescents (MINI KID) [12, 13]. The MINI KID was chosen because of its diagnostic relevance based on DSM-IV and ICD 10 criteria. It has also been used previously in child mental health surveys in Uganda [14, 15]. Depending on reported symptoms and observations, the appropriate diagnosis e.g. attention deficit and hyperactivity disorder (ADHD), depression or conduct disorder was made. Children with an observed difficulty that did not fulfil a specific mental health disorder criterion were described as having behaviour difficulties only. In both cases referral was made to local services for care.

### Brain magnetic resonance imaging

Brain MRI were performed on selected cerebral malaria exposed children with mental health sequelae and two randomly selected children without sequelae (behaviour difficulty, mental health disorder or neurologic deficits). Images were acquired using a Phillips Achieva 1·5 Tesla 16 channel A series machine under sedation using chloral hydrate. MRI sequences included T1 (3D multi plannar isotropic scans; FOV 240 mm, slice thickness 1 mm, TE 4.6, TR = 25, slice gap 0.4 mm and flip angle = 30) and T2 weighted scans (2D scans; TE110, shortest TR, slice thickness 4 mm, slice gap 1 mm, and flip angle = 90), Fluid Attenuated Inversion Recovery (FLAIR) and diffusion weighted imaging (DWI). Qualitative analyses of the images were performed by a radiologist who was not aware of the child’s diagnosis for global and focal changes and lesions.

### Statistical analysis

Data analysis was performed using STATA version 12 (STATA Corp, Tx). Proportions of patients with specific behaviour difficulties and mental health disorders between groups were compared using Chi square analysis, with a Bonferroni correction for multiple comparisons. Multivariate logistic regression was used to assess clinical and demographic risk factors for mental health disorders.

## Results

### General characteristics

Parents of 392/430 (91.1 %) invited children accepted participation. Ten children were excluded; two with developmental issues that may have preceded the study, but were previously unreported, one who had developed severe HIV/AIDS, and seven who did not complete the assessments. Parents of two other children (one cerebral malaria-exposed and one community child) withdrew consent. Of the remaining 380 participants, 173 were had cerebral malaria, 99 had severe malaria anaemia and 108 were community children. One hundred and three children (59.5 %) with cerebral malaria, 62 (62.6 %) with severe malaria anaemia, 62.6 % and 42 (38.9 %) community children were male. The mean (SD) ages of the three groups were: cerebral malaria 4.1(2.0) years; severe malaria anaemia 3.4(1.5) years and community controls 4.3(1.9) years while the median time from exposure to assessment was 21 (IQR 12–24) months.

### Behaviour difficulties on screening with the SDQ

**Fig. 1:**
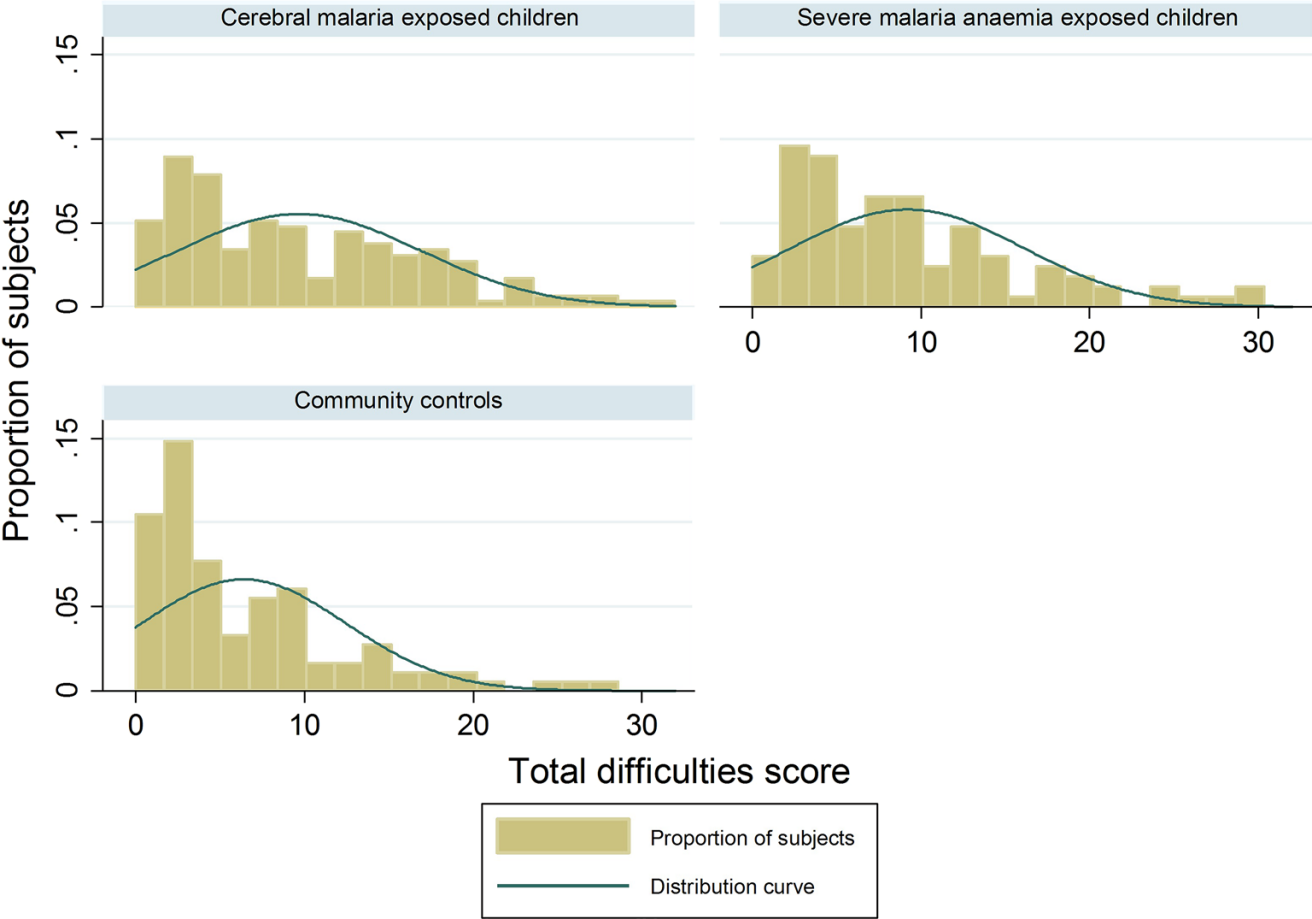
This is a distribution of total difficulties scores on the SDQ by study group

The spread of the total difficulties score is described in Fig. 1 and in Table 1. Overall, 55/380 (14.5 %) participants had abnormal total difficulties scores. Abnormal scores were more frequent in children with cerebral malaria (18.5 %) than community children (7.4 %, *p* = 0.02, Table 2) but were almost as frequent in children with severe malaria anaemia (15.3 %). However, differences in the frequency of abnormal scores in children with severe malaria anaemia as compared to community children did not achieve statistical significance possibly due to power issues as the number of children tested was smaller than those with cerebral malaria.

**Table 1:** Spread of total difficulties scores of the SDQ in children exposed to cerebral malaria compared to children exposed to severe malaria anaemia only and to community controls

Range of total difficulties scores	Cerebral malaria, n = 173 (%)	Severe malaria anaemia, n = 99 (%)	Community controls, n = 108 (%)	Total for the range of scores, n = 380 (%)
Normal scores, 0–13	121 (69.4)	78 (78.8)	93 (86.1)	292 (76.4)
Borderline scores, 14–16	20 (11.6)	6 (6.1)	7 (6.5)	33 (8.7)
Abnormal scores, 17 or higher	32 (18.5)	15 (15.2)	8 (7.4)	55 (14.5)

**Table 2:** Abnormal SDQ scores in children exposed to severe malaria compared to community children

Abnormal SDQ scores by difficulty groups	Cerebral malaria (CM), n = 173 (%)	Severe malaria anaemia (SMA), n = 99 (%)	Community children CC), n = 108 (%)	*P* ^a^ value (CM vs CC)	*P* ^a^ value (SMA vs CC)	*P* ^a^ value (CM vs SMA)
Abnormal total difficulties score (scores 17–40)	32 (18.5 %)	15 (15.2 %)	8 (7.4 %)	0.02	0.15	0.97
Abnormal emotional score (scores 5–10)	37 (21.4 %)	18 (18.2 %)	13 (12.0 %)	0.09	0.43	0.99
Abnormal conduct score (scores 4–10)	37 (21.4 %)	22 (22.2 %)	11 (10.2 %)	0.03	0.04	0.99
An abnormal hyperactivity score (scores 7–10)	27 (15.6 %)	11 (11.1 %)	4 (3.7 %)	0.004	0.08	0.61
Abnormal peer relations score (scores 4–10)	38 (22.0 %)	16 (16.2 %)	14 (13.0 %)	0.12	0.99	0.50
Abnormal pro-social score (scores 0–4)	14 (8.1 %)	6 (6.1 %)	5 (4.6 %)	0.522	0.99	0.99

^a^ χ^2^ test or Fisher’s exact test with Benferroni correction

With the SDQ subscales, children with cerebral malaria had abnormal scores more frequently detected in conduct and hyperactivity scores than community children (Table 2). Children with severe malaria anaemia also had more frequent abnormal conduct scores than community children, and differences in hyperactivity scores between children with severe malaria anaemia and community children approached significance (Table 2).

### Specific behaviour difficulties and mental health disorders

All 55 children with abnormal total difficulties scores (32 cerebral malaria, 15 severe malaria anaemia and eight community children) had a detailed mental health interview. Using the MINI KID, 18 children with cerebral malaria (10.4 %) and four children with severe malaria anaemia (4.0 %) were defined to have a mental health disorder as compared to two community children (1.8 %, *p* = 0.01 for CM compared to CC). In contrast, behavioural disorders only were seen in six children with cerebral malaria (3.5 %), four children with severe malaria anaemia (4.0 %) and three community children (2.8 %, *p* not significant for any comparison), Table 3.

**Table 3:** Behaviour difficulties and mental health disorders after severe malaria

Behaviour difficulty or mental health disorder	Cerebral malaria, n = 173, (%)	Severe malaria anaemia, n = 99, (%)	Community controls, n = 108, (%)	*p* ^a^ value (CM vs CC)	*p* ^a^ value (SMA vs CC)	*p* ^a^ value (CM vs SMA)
Behaviour difficulty only	6 (3.5)	4 (4.0)	3 (2.8)	0.99	0.99	0.99
Mental health disorder	18 (10.4)	4 (4.0)	2 (1.8)	0.01	0.69	0.13

^a^χ^2^ test or Fisher’s exact test with Benferroni correction

The most common mental health disorders among the 18 cerebral malaria-exposed children were ADHD (n = 7), conduct disorder (n = 7) and oppositional defiant disorder (n = 7). Others were depression (n = 4), and social (n = 1), separation (n = 1) or generalized (n = 1) anxiety disorder (Table 3). No child had autism spectrum disorder. Behaviour disorders included outbursts of anger and aggression, antisocial behaviour, attention difficulties and non-persistent depressive symptoms. Others were secondary enuresis and temper tantrums. The onset of symptoms of all the behavioural and mental health disorders was within 3–12 months of discharge. Among the four children with severe malaria anaemia, conduct disorder (n = 2), oppositional defiant disorder (n = 2), depression (n = 1) and ADHD (n = 1) were seen (Table 4). Mental health disorders in the two community controls were depression in one child and anxiety disorder in the second while the three with behaviour difficulties had anti social behaviour and emotional problems.

**Table 4:** Long-term mental health disorders and brain MRI findings in children with cerebral malaria

ID	Exposure	Gender female/male	Age on exposure, years	Parents concerns	Specific mental health disorder on DSM-IV criteria	Brain MRI findings
1	Cerebral malaria	Male	1.9	Episodic aggressive behaviour, wets his bed, restless and inattentive	ADHD (inattentive type) also has anxiety and depressive symptoms	Brain MRI not done
2	Cerebral malaria	Female	2.2	Extremely quiet; plays alone and destroys property; bed wetting	Major depression (current)	Normal brain MRI
3	Cerebral malaria	Female	5.7	Running away from home, often leaves the classroom and moves around	Conduct disorder	Brain MRI not done
4	Cerebral malaria	Male	5.8	Aggressive behaviour, inattentive, excessive talking, conduct problems	ADHD (inattentive type) conduct disorder	Bilateral punctuate (but few) white matter, high T2 signal foci measuring 1.5–2.0 mm in the parietal regions regions of the cerebral hemispheres close to the vertex. No restriction of water diffusion (on DWI)
5	Cerebral malaria	Male	3.0	Shy, low self esteem	Social phobia (social anxiety disorder)	Normal brain MRI
6	Cerebral malaria	Male	4.0	Fighting, aggressive behaviour, anger, lies, poor concentration at school and spending nights outside home	Conduct disorder ADHD (inattentive type)	Brain MRI not done
7	Cerebral malaria	Male	3.9	Fears being alone; cries often, aggressive to peers and easily confused	Separation anxiety disorder	Generalised widening of sulcal spaces and sylvian fissures and numerous bilateral hyperintensities in the grey mater and subcortical regions of the frontal, occipital and temporal lobes
8	Cerebral malaria	Male	6.9	Irritable, aggressive towards siblings, sucking thumb	Adjustment disorder (current)	Bilateral hyper-intensities around the frontal horns of the lateral ventricles—largest measuring 5-7 mm and also the temporal and occipital horns. No oedema or restricted diffusion on DWI
9	Cerebral malaria	Female	2.0	Feeding problems, weight loss, fearful, quiet and plays alone, nightmares. Separated from parents	Major depression (current)	Normal brain MRI
10	Cerebral malaria	Male	2.7	Defiant and indifferent—not caring attitude, labile mood, disorganised, misplaces objects	Oppositional defiant disorder	Brain MRI not done
11	Cerebral malaria	Male	3.0	Loss of interest; poor concentration and irritability	Major depression (current)	Normal brain
12	Cerebral malaria	Male	2.6	Hyperactive aggressive	Oppositional defiant disorder	Normal brain
13	Cerebral malaria	Female	2.1	Irritable, aggressive, self injurious behaviour, head banging, excessive crying, temper tantrums, regression in speech	Oppositional defiant disorder Also has epilepsy, bilateral hyperreflexia and upgoing babinksi reflexes and learning disability	High signal white mater intensities on T2 weighted imaging and FLAIR (iso-intense on T1 WI)
14	Cerebral malaria	Male	3.2	Violent and aggressive behaviour stealing	ADHD Conduct disorder Has severe sequelae with visual, hearing, speech impairment, epilepsy and learning difficulties	Marked global brain atrophy involving the cerebral hemispheres, midbrain, brainstem and cerebellum and reduced grey-white mater differentiation. Focal encephalomalacia in both occipital lobes and demyelination with extensive T2 W and T2FLAIR white mater peri-ventricular hyperintensities and in the right external capsule, cerebellae penducle and cerebellar hemispheres
15	Cerebral malaria	Female	7.1	Fearfulness, very slow, irritable, quiet, frequent headaches	Major depression (current) also has some anxiety symptoms and learning difficulties	Normal brain
16	Cerebral malaria	Male	6.9	Worsening episodes of aggressive behaviour; fighting, resists change, forgetful, cries often, throws stones; enuresis	ADHD inattentive type; conduct disorder	Bilateral hyper-intensities around the parietal and occipital areas—largest measuring 2–3.7 mm and also the temporal and occipital horns. No oedema or restricted diffusion on DWI
17	Cerebral malaria	Male	3.5	Wets himself during day; Isolates himself and hides, quiet most of the time	Separation anxiety disorder. Major depression (current)	Bilateral punctuate white mater high T2 signal foci (hypo-intense on T1 WSE) measuring 1.5-2.0 mm in the parietal regions. No restriction of water diffusion on DWI
18	Cerebral malaria	Female	9.0	Forgetfulness, fearful and reduced concentration; declining academic performance and often punished	ADHD combined	Normal brain

*ADHD* attention deficit hyperactivity disorder; *ODD* oppositional defiant disorder

### Neurologic sequelae

On discharge from hospital, neurologic deficits were documented in 41/173(23.1 %) children with cerebral malaria. These included motor difficulties (weakness, hyperreflexia and global hypotonia) in 35 (20.2 %) children, visual (9/173, 5.2 %), hearing (1/173, 0.6 %) and speech and language (17/173, 9.8 %) impairments. In addition, gait problems and ataxia probably a result of an incomplete recovery from a severe illness were observed in 25/173 (14.4 %). At the time of follow-up assessment, these deficits had resolved except in five children (2.9 %) who initially had severe motor deficits.

### Brain MRI findings

**Fig. 2:**
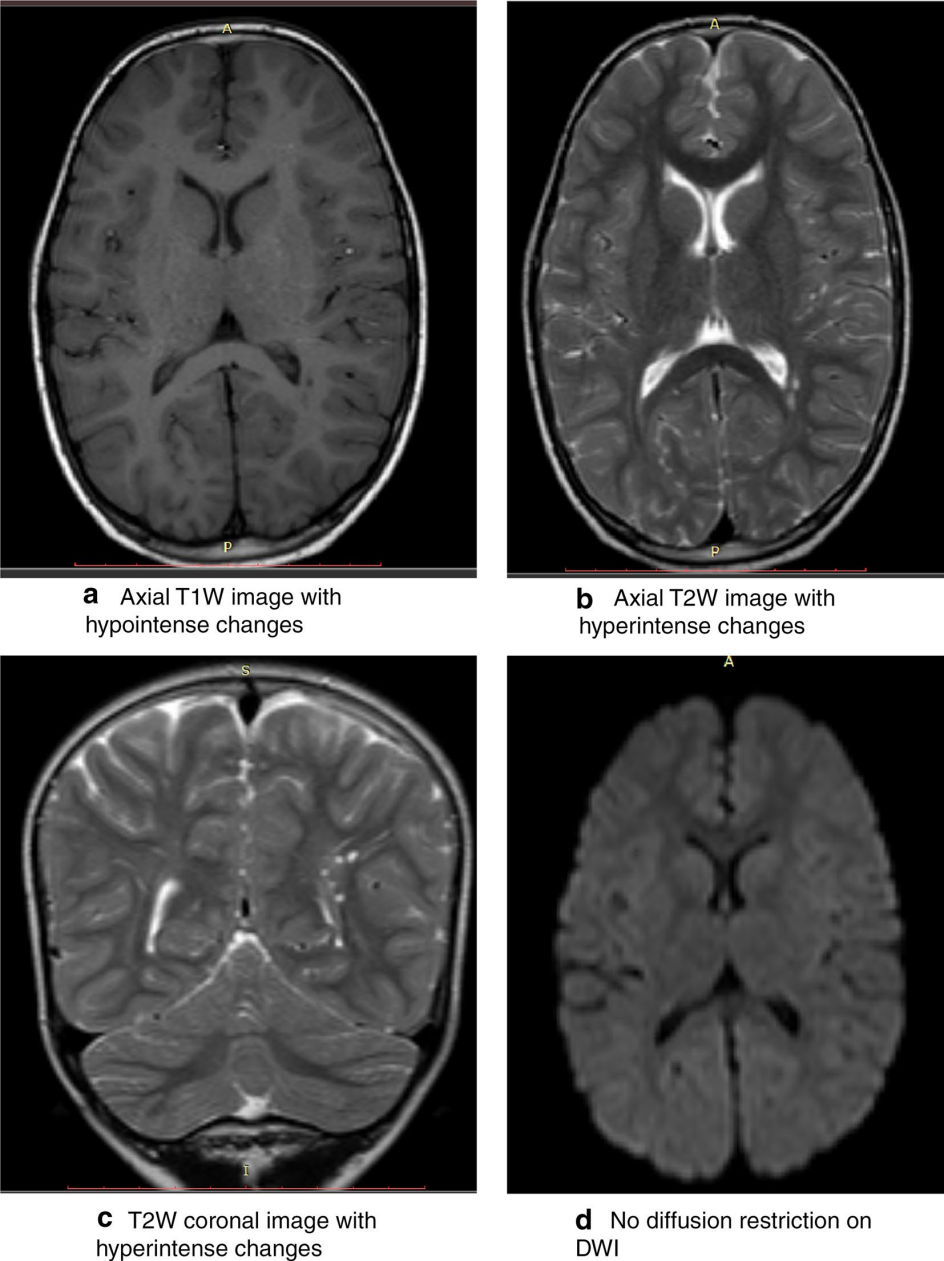
This is a set of brain MRI images of a 7 year old boy who suffered cerebral malaria at the age of 4 years. On discharge he only had hyperreflexia. His neurologic examination 3 years later was normal but the mental health assessment demonstrated oppositional defiant disorder. The brain MRI had bilateral wide spread punctate T1 weighted (T1W) hypointensities (**a**) and T2W hyperintensities (**b**) and (**c**) in the *white mater* and most marked in the parietal and temporal areas. There are associated with periventricular hyperintensities around the posterior horns of the lateral ventricles the largest of which measures 6 mm and widening of the sulcal spaces in the frontal and parietal lobes. The lesions exhibit no diffusion restriction on diffusion weighted imaging (DWI), (**d**). The changes suggest bilateral wide spread small vessel ischemia and cerebral atrophy. *T1W* T1 weighted imaging, *T2W* T2 weighted imaging, *DWI* diffusion weighted imaging

**Fig. 3:**
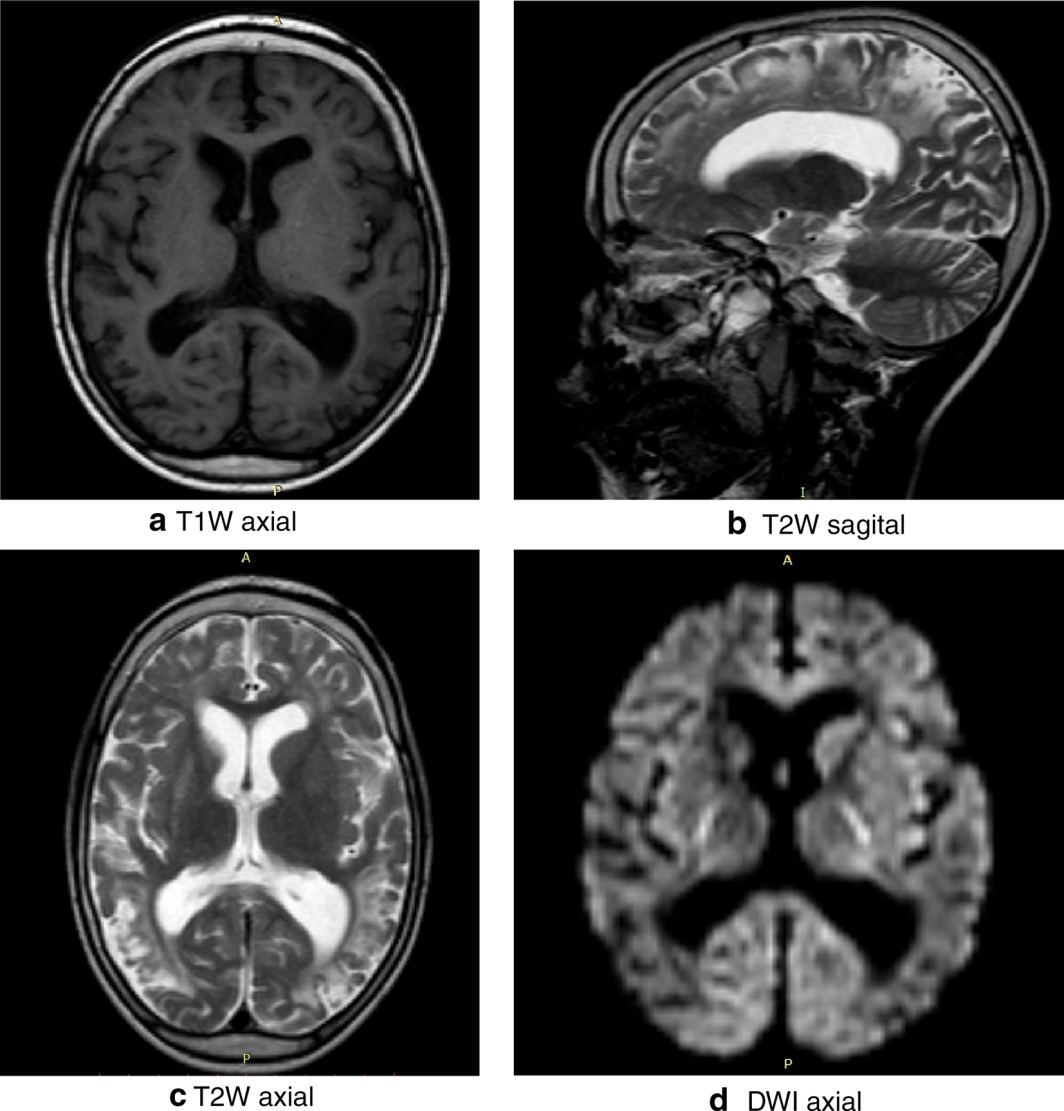
This is a set of brain MRI images of a 5 years old boy who suffered cerebral malaria at the age of 3 years and 3 months. On discharge he had severe neurological sequelae with motor, visual, hearing, and speech deficits and later developed epilepsy. At the time of imaging 20 months later, the visual, speech and motor difficulties had markedly improved but he had developed conduct problems and attention deficit and hyperactivity disorder with violent and aggressive behaviour. The images show generalized widening of sulcal spaces and sylvian fissures in both cerebral hemispheres. There are numerous bilateral T1 W hypointense foci showing confluence in some areas (**a**); hyper-intense foci in the *grey mater* and sub cortical regions of the frontal occipital and temporal lobes on T2 W (**b** and **c**) and fluid fluid-attenuated inversion recovery (FLAIR) images. There was no restriction of water diffusion on DWI, (**d**)

Brain MRI was performed for 16 cerebral malaria-exposed children including 14 with mental health disorders (with or without neurologic deficits) and two children who survived without sequelae. The MRI findings in the 14 children with mental health disorders are summarized in (Table 3). The two children who survived without sequelae and seven of 14 with mental health disorders had MRI images that were qualitatively read as normal. Among the seven patients with mental health disorders and abnormal MRI, a common set of findings on the imaging was multiple patchy, punctuate and often bilateral hyper-intense foci on T2 weighted and FLAIR images measuring 1.5–3.0 mm in diameter. The lesions were iso or hypo-intense on T1 weighted imaging. The severity of involvement ranged from mild to severe (extensive). There was no oedema or restriction of water diffusion on DWI. These lesions were seen in peri-ventricular areas and around the frontal, temporal or occipital horns of the lateral ventricles—the largest lesion measured 7.0 mm. Examples of these lesions, from the MRI of a child with oppositional defiant disorder and of a child with attention deficit and hyperactivity disorder and conduct disorder are shown in Figs. 2, 3. Together, these features suggest small vessel ischaemia and micro-infarctions. These findings were not seen in either of the two children with depression. In the two children with persisting neurologic sequelae, cortical atrophy with generalized widening of sulcal spaces and sylvian fissures and hyper-intense lesions in the grey mater and sub-cortical regions were also observed.

### Risk factors for mental health disorders after severe malaria

The relationships between mental health disorders in children following severe malaria and age at exposure as well as the following known prognostic markers of severe malaria were examined: presentation with profound coma, status epilepticus, malaria retinopathy or hypoglycaemia, the peripheral blood parasite density, haemoglobin and lactic acid levels, the total duration of coma and duration of hospitalization. Profound coma was defined as Blantyre coma score of ≤1 (out of 5) or Glasgow coma score of ≤6. Children who developed mental health disorders were more likely to have experienced seizure recurrences during hospitalization, a slower recovery from coma, and to have been discharged with neurologic sequelae, Table 5. The median total coma duration in those with mental health disorders was 72 (IQR 36.0–115.0) h compared to 48 (IQR 28.5–78.7) h in those without, *p* = 0.039. Although there were no differences in the proportions of patients with and without mental health disorders by gender, there was a trend for males with such disorders to exhibit externalizing problems as opposed to females who appeared to exhibit internalizing problems. The numbers were however small to explore these differences further. Multivariate logistic regression was then performed for all variables with a *p* value of <0.2. Discharge with neurologic sequelae, adjusted OR 4.09 (95 % CI 1.60–10.5), *p* = 0.003 and seizure recurrences during hospitalization, adjusted OR 2.80 (95 % CI 1.13–6.97), *p* 0.026, were independently associated with an increased risk of mental health disorders.

**Table 5:** Factors associated with behaviour difficulties and mental health disorders following severe malaria

Demographic and clinical features	Cerebral malaria exposed children, N = 173			Severe malaria anaemia exposed children, N = 99		
	With mental health disorder, n = 18	No mental health disorder, n = 155	*p* ^a^ value	With mental health disorder, n = 4	No mental health disorder, n = 95	*p* ^a^ value
Gender, male (%)	12 (66.7)	91 (58.7)	0.515	2 (50.0)	60 (63.2)	0.594
Age on exposure, mean (SD) yr	4.2 (2.1)	4.0 (2.0)	0.693	3.2 (1.9)	3.4 (1.4)	0.812
Duration of fever prior to hospitalization, median (IQR) days	3 (2.5)	3 (2.4)	0.149	3. (3.4)	4 (3.5)	0.695
Profound coma (BCS ≤ 1 or GCS ≤ 6) on admission	7 (38.9)	39 (25.2)	0.212	–	–	–
Seizure recurrences during hospitalization, (%)	13 (72.5)	79 (51.0)	0.087	–	–	–
Admission Hb, mean (SD) in g/dl	7.8 (2.1)	7.1 (2.1)	0.150	4.6 (1.2)	4.6 (2.0)	0.841
Admission lactate, mean (SD)	4.1 (4.4)	4.6 (3.4)	0.588	6.0 (3.0)	5.2 (3.6)	0.694
Hypoglycaemia (blood glucose <2.2 mmol/L) on admission	3 (16.7)	8 (5.2)	0.058	0 (0)	4 (4.4)	0.543
Presence of malaria retinopathy, %	10 (55.8)	103 (66.5)	0.358	–	–	–
Total duration of coma, median (IQR) hours^b^	72.0 (36.0, 115)	48.0 (28.5, 78.7)	0.039	–	–	–
Neurologic sequelae on discharge, %	9 (50.0)	32 (20.7)	0.006	–	–	–

^a^Student’s t test for age, Wilcoxon rank-sum for other continuous variables, χ^2^ or Fisher’s exact test as appropriate for categorical variables

^b^Time from onset of coma to regaining full consciousness in hours

## Discussion

This study set out to describe behaviour difficulties and mental health disorders following cerebral malaria and the structural brain imaging changes associated with these sequelae, determine the early predictive features and examine if children exposed to other complications of severe malaria and in particular, severe malaria anaemia, have similar increased risk. The study found that cerebral malaria but not severe malaria anaemia was associated with an increased risk of mental health disorders. Externalizing problems (conduct, oppositional defiance and attention deficit hyperactivity disorders) were the most common problems. In affected children, brain imaging suggested that small vessel ischaemic injury is linked to these difficulties.

This is the first study to systematically document the mental health sequelae of more than one form of severe malaria in children and to have combined clinical assessments with diagnostic imaging. The data suggests that (1) over 10 % of cerebral malaria exposed children may have long-term mental health sequelae; (2) the sequelae, especially the externalizing behaviours of ADHD, conduct and oppositional defiant disorders, may be a consequence micro-vascular ischaemic neural injury and develop within 12 months of exposure to cerebral malaria. There was no relationship between gender, age on exposure, peripheral blood parasite density on hospitalization, haemoglobin and lactic acid levels and these sequelae. However, features of a more severe disease—seizure recurrences during hospitalization and discharge with neurologic sequelae, were independently associated. The slower recovery from coma may also be a consequence of the suggested ischaemic brain injury in affected individuals. The imaging data here is similar to that reported in Malawi except for the clearer evidence of small vessel ischaemia [16]. Three patterns are recognizable; normal brain MRI, small vessel ischaemic brain injury and severe brain injury with extensive cortical atrophy. No detailed volumetric analysis was however performed and the effect cerebral cortical volume loss on function was not determined. However, although the study appears to suggest the mental health sequelae as a direct consequence of the brain injury, it could also be that some is a consequence of stress associated with suffering a serious disease. Additional data and analysis would be helpful to delineate te different contributions.

Between the years 1994 and 2004, it was estimated that annually, there were a minimum 2.8 million hospitalizations with severe malaria in Africa [17]. About 10 % of this hospitalization is due to cerebral malaria [3]. Although the burden of malaria has declined since then, with mental health disorders being described in about 10 % of child survivors of cerebral malaria [3], our study suggests that since 1994, cerebral malaria may be responsible for severe mental health problems in at least 30,000 children annually in the malaria endemic regions of Africa. The need to work towards malaria eradication in the continent cannot be overstated.

Currently, there are no interventions for children who develop these problems and no programmes for follow-up to identify affected individuals. With up to 25 % of cerebral malaria survivors developing long-term neuro-cognitive deficits [5] and 10 % showing evidence of mental health disorders, it is more urgent than ever to put in place programmes of post hospitalization follow-up care for exposed children. However, with the limited trained mental health specialists and tools to identify these deficits, there is also need to develop or adapt simple and appropriate screening tools to identify patients at risk.

This study has some limitations; first, the study did not systematically examine effects of the home environment (e.g. parenting and child rearing) on the outcomes which could have contributed to the difficulties. Secondly, the psychometric properties, such as internal consistency, of the instruments used are not reported although all have previously been used in East Africa. Third, the pre-morbid state of the participants is unknown. This study, therefore, cannot be absolute in its attribution of causation.

## Conclusions

In addition to high mortality and long-term neuro-disability, cerebral malaria predisposes children to mental health disorders, probably as a result of ischaemic neural injury. There is urgent need to put in place programmes for follow-up, identification and interventions for these children.
